# An Overview of Structurally Modified Glycyrrhetinic Acid Derivatives as Antitumor Agents

**DOI:** 10.3390/molecules22060924

**Published:** 2017-06-02

**Authors:** Bing Xu, Gao-Rong Wu, Xin-Yu Zhang, Meng-Meng Yan, Rui Zhao, Nan-Nan Xue, Kang Fang, Hui Wang, Meng Chen, Wen-Bo Guo, Peng-Long Wang, Hai-Min Lei

**Affiliations:** School of Chinese Pharmacy, Beijing University of Chinese Medicine, Beijing 100102, China; weichenxubing@126.com (B.X.); gaorongwu09@163.com (G.-R.W.); xinyums@126.com (X.-Y.Z.); yanmengmeng@bucm.edu.cn (M.-M.Y.); zr1012@bucm.edu.cn (R.Z.); m18810612758@163.com (N.-N.X.); 18364166994@163.com (K.F.); 15652387323@163.com (H.W.); 18702267252@163.com (M.C.); wb_guo@126.com (W.-B.G.)

**Keywords:** glycyrrhetinic acid, overview, structural modification, antitumor

## Abstract

Glycyrrhetinic Acid (**GA**), a triterpenoid aglycone component of the natural product glycyrrhizinic acid, was found to possess remarkable anti-proliferative and apoptosis-inducing activity in various cancer cell lines. Though **GA** was not as active as other triterpenes, such as betulinic acid and oleanolic acid, it could trigger apoptosis in tumor cells and it can be obtained easily and cheaply, which has stimulated scientific interest in using **GA** as a scaffold to synthesize new antitumor agents. The structural modifications of **GA** reported in recent decades can be divided into four groups, which include structural modifications on ring-A, ring-C, ring-E and multiple ring modifications. The lack of a comprehensive and recent review on this topic prompted us to gather more new information. This overview is dedicated to summarizing and updating the structural modification of **GA** to improve its antitumor activity published between 2005 and 2016. We reviewed a total of 210 **GA** derivatives that we encountered and compiled the most active **GA** derivatives along with their activity profile in different series. Furthermore, the structure activity relationships of these derivatives are briefly discussed. The included information is expected to be of benefit to further studies of structural modifications of **GA** to enhance its antitumor activity.

## 1. Introduction

Natural products have played a highly significant role in the medicine discovery and development processes and many useful medicines were developed from plant sources [1]. This was particularly evident in the area of cancer treatment, where over 60% of current antitumor drugs, such as vinblastine, etoposide and paclitaxel, originated from Nature [2].

**Figure 1:**
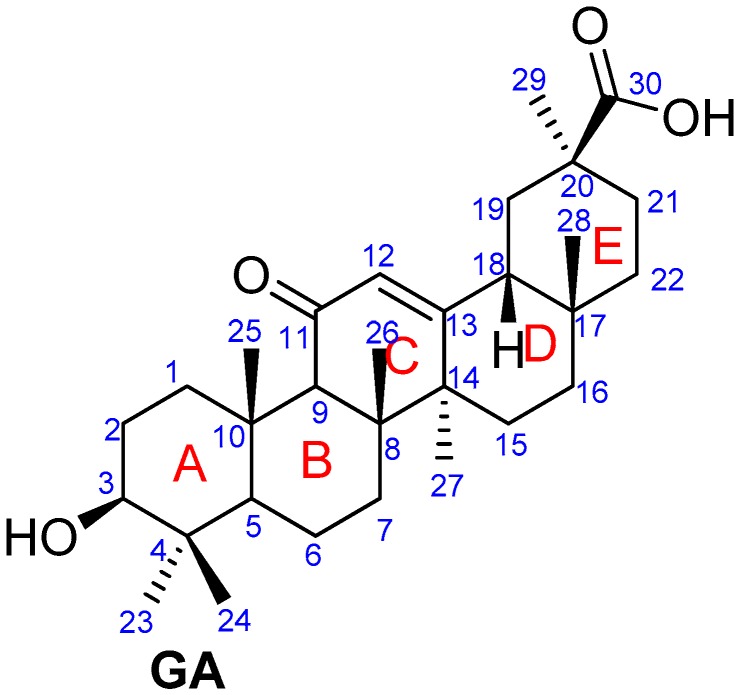
Structure of glycyrrhetinic acid.

Glycyrrhetinic acid (**GA**, Figure 1) is a triterpenoid aglycone component of the natural product glycyrrhizinic acid (**GL**), which is abundant in licorice root [3]. **GA** was proved to possess a variety of remarkable biological activities, including anti-inflammatory [4,5], antiviral [6,7], hepato- protective [8,9], and antitumor properties [10,11]. **GA** is highly regarded for its remarkable antitumor activities, whereby it shows significant cytotoxic activity against a broad variety of different cell types in vitro, for example non-small cell lung cancer cells [11], pituitary adenoma cells [12], human hepatocellular carcinoma cells [13], prostate cancer cells [14] and glioblastoma cells [15]. It also exhibits noteworthy activity in various experimental cancer models in vivo [16,17], and it is known to trigger apoptosis in tumor cell lines [14,18,19]. Some experimental reports have indicated that **GA** triggered apoptosis via the mitochondrial pathway through the collapse of mitochondrial membrane potential, the accumulation of the cytosolic cytochrome c and the activation of caspase-9 and caspase-3 [19,20].

The remarkable antitumor activity of **GA** has been the focus of researchers worldwide. However, because **GA** can inhibit type 2 11ß-hydroxysteroid dehydrogenase (11ß-HSD2), administrating **GA** at a high dose for a long time often causes pseudoaldosteronism, which is characterized by hypertension, hypokalemia and other adverse clinical effects [21,22,23]. Studies on using **GA** as a scaffold to develop new low-toxicity and high-effectivity antitumor agents have attracted much attention, and a number of structural modifications of **GA** were carried out and some reports of novel **GA** derivatives as antitumor agents have been published [24,25,26]. This overview is dedicated to summarizing and updating four aspects of the structural modification of **GA** leading to antitumor agents published between 2005 and 2016, including modifications at the ring-A, ring-C, ring-E and multiple ring modification. We have compiled the most active **GA** derivatives along with their activity profile in different series. Furthermore, the structure activity relationships of these derivatives are briefly discussed.

## 2. Four Aspects of the Structural Modifications of Glycyrrhetinic Acid

In the past few years, plenty of researchers around the world have designed and synthesized series of **GA** derivatives as potential antitumor agents. Most reports about the chemical and structural modifications of **GA** were focused on the specific functional groups of the A, C, and E rings, as these three rings contain three functional groups which are the most suitable for modification: a hydroxyl group at C-3 in ring-A, an α,β-unsaturated carbonyl function located in ring-C at C-11 and a carboxyl group at C-30 on ring-E. Meanwhile, studies on the skeleton ring architecture modification of this pentacyclic triterpene are increasing too, hence, the modifications of **GA** to produce novel antitumor agents can be classified into four styles, including structural modifications at ring-A, at ring-C, at ring-E and at multiple ring modifications.

### 2.1. Structural Modifications on Ring-A

#### 2.1.1. Structural Modifications at the C3-OH in Ring-A

The structural modifications at the C3-OH group of **GA** are very common. For example, it could be converted into an oxime group, a carbonyl group and a 3-oxo group. However, in order to change the polarity pattern or improve the antitumor activity of **GA**, the C-30 carboxyl group was often esterified too.

**Scheme 1:**
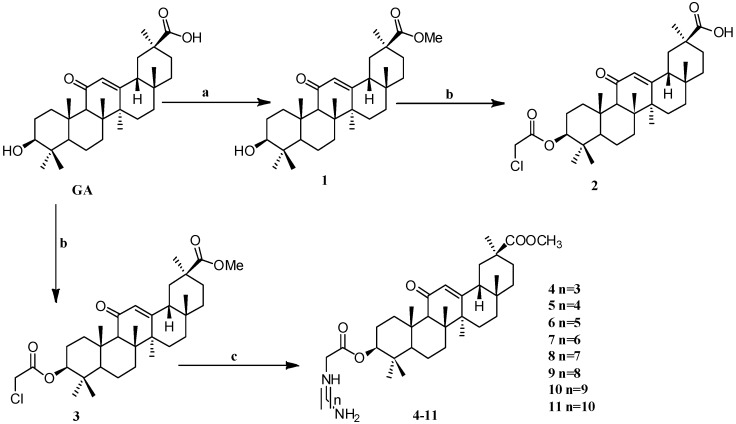
Synthesis of the **GA** amino alkyl derivatives **1**–**11**. *Reagents and conditions*: (a) K_2_CO_3_,CH_3_I, DMF, 24 h, 25 °C; (b) ClCH_2_COCl, Et_3_N, THF (or CH_2_Cl_2_), 25 °C, 12 h; (c) H_2_N-(CH_2_)*_n_*-NH_2_, DMF, K_2_CO_3_, 12 h, 25 °C.

It was reported that changing the polarity pattern of **GA** might be an advantage in obtaining better cytotoxicity. Based on this, different C-3 amino alkyl derivatives of **GA** (compounds **4**–**11**, Scheme 1, were synthesized by Csuk et al. [27]. The antitumor activity of these derivatives was tested in a panel of 15 human cancer cell lines by a SRB assay. In the SRB assay, all of the amino compounds **4**–**11** showed significantly improved activity compared with **GA**. Among them, it could be observed that a diaminohexyl chain with seven carbon atoms was the most active derivative, about 60 times more so than **GA**. The antitumor activity was changed with the change of the carbon number. The results also showed that the esterification at C-30 (compound **3**, Scheme 1) could improve the antitumor efficacy compared with compound **2**. The same result could be found from previous findings and parallel results [28,29,30,31,32]. Besides, the introduction of nitrogen-containing substituents to the ring-A seemed to improve the anti-proliferative effect of **GA** derivatives. The cytotoxicity (IC_50_ values in μmol) of **1**–**11** in a panel of various cancer cell lines is summarized in Table 1.

**Scheme 2:**
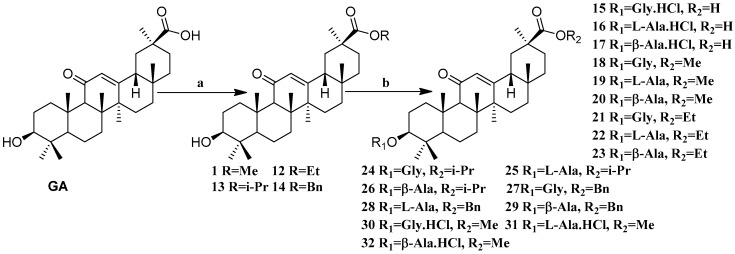
Synthesis of the **GA** amino acid derivatives **12**–**32**. *Reagents and conditions*: (a) K_2_CO_3_, alkyl halides, DMF, 24 h, 25 °C; (b) These compounds were synthesized by DCC mediated esterification of N-Boc protected amino acids followed by their deportation using TFA in dry DCM (for the amines) or by treating them with dry HCl gas in DCM (for the ammonium hydrochlorides).

Similarly, in order to change the polarity pattern of **GA**, Schwarz et al. [33] prepared a series of novel derivatives **12**–**32** by introducing an extra amino group into C-3 and esterifying at C-30 (Scheme 2). These derivatives showed a higher antitumor activity and a better selectivity towards tumor cells compared with **GA** on 15 different human tumor cell lines and mouse embryonic fibroblasts (NiH3T3). Compound **24** substituted with glycine and esterified with an *i*-propyl moiety was the most active compound. As discussed above for antitumor activity, in this case, the esterification at C-30 also resulted in improved activity against tumor cell lines compared with **GA**. The most active compound among the C-30 ester derivatives was the benzyl ester (compound **14**) showing IC_50_ value between 6.15–23.82 µM. The decrease of the IC_50_ value paralleled the size and lipophilic character of the alkyl chain of the esters. From the SAR of these compounds, it was concluded that the introduction of an extra amino acid moiety at C3-OH or an alkyl group at C30-COOH could enhance the antitumor activity. There seemed to be no effect by adding a stereogenic center in the side chain according to the results. Besides, the amines and their respective ammonium salts might be considered bioequivalent in biological activity. The cytotoxicity (IC_50_ values in µM) of **12**–**32** in a panel of various cancer cell lines is summarized in Table 2.

**Scheme 3:**
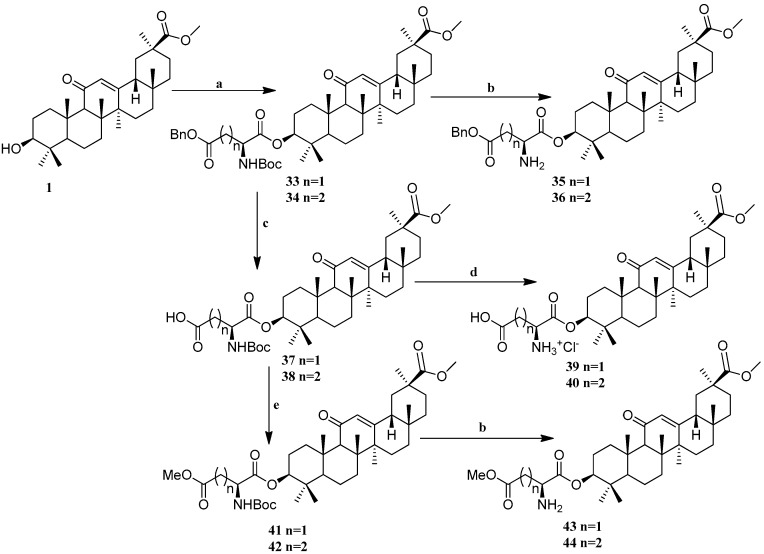
Synthesis of the **GA** amino acid derivatives **33**–**44**. *Reagents and conditions*: (a) DCC, DMAP, Boc-Asp(OBzl)OH or Boc-Glu(OBzl)OH, DCM, 12 h, 25 °C; (b) TFA, DCM, 12 h, 25 °C; (c) NH_4_^+^HCO_2_^−^, Pd/C (10%), THF/MeOH, 12 h, 25 °C; (d) HCl (gas), DCM, 12 h, 25 °C; (e) CH_3_I, K_2_CO_3_, DMF, 2 h, 25 °C.

In subsequent research Csuk et al. conducted another study in a similar manner, producing a series of derivatives **33**–**44** substituted with aspartic and glutamic acid (Scheme 3) [34]. The glutamic acid derivative **36** with a benzyl-protected side chain was the most active derivative among this series, showing an IC_50_ value between 1.27–2.33 µM. Meanwhile, compound **36** displayed an extraordinary selectivity (Mean F = 23) in comparison with other compounds. The derivatives carrying a free amino group and an unprotected carboxylic group such as compounds **39** and **40** turned out to be inactive (IC_50_ > 100 µM). The cytotoxicity (IC_50_ values in µM) of **33**–**40**, **43**, **44** in a panel of various cancer cell lines is summarized in Table 3.

**Scheme 4:**
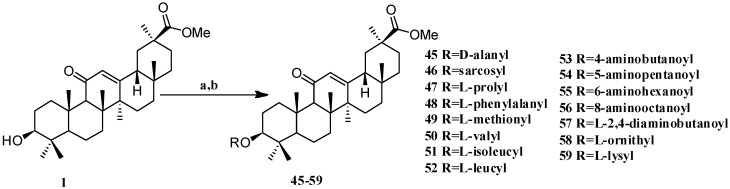
Synthesis of the **GA**–**Me** (**GA** methyl ester**)** amino ester derivatives **45**–**59**. *Reagents and conditions*: (a) Boc-amino acids, DCM, DMAP, DCC, 12 h, 25 °C; (b) TFA in DCM, 12 h, 25 °C, or HCl (gas) in DCM, 12 h, 25 °C.

As mentioned, introduction an extra amino group into C-3 and esterification at C-30 could improve the antitumor activity of **GA** derivatives. To further increase the cytotoxicity and improve the selectivity, some other amino acid derivatives of glycyrrhetinic acid **45**–**59** (Scheme 4) were designed and synthesized in a similar way by Csuk et al. [35]. The derivatives possessing short side chains like the alanyloxy or sarcosyloxy moiety, turned out to exhibit higher cytotoxic activity, for example, compound **46** showed IC_50_ values between 1.83 and 3.42 µM. However compounds with a more lipophilic side chains, such as compound **50**, **51** showed decreased cytotoxic effects compared with **GA**–**Me** in the SRB assay. These results indicated that the structure of the amino acid side chain affected the cytotoxicity most. The cytotoxicity (IC_50_ values in µM) of **45**–**59** on a panel of various cancer cell lines is summarized in Table 4.

**Scheme 5:**
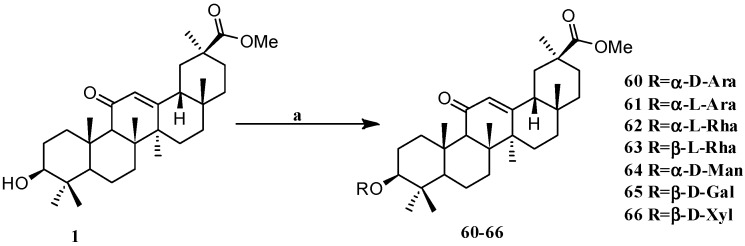
Synthesis of the **GA** glycosides derivatives **60**–**66**. *Reagents and conditions*: (a) Sugar trichloro acetimidate, TMSOTf, DCM, −70 °C–25 °C, 2 h.

It was reported that the introduction of an extra hydrophilic sugar moiety into betulinic acid could increase its cytotoxicity [36]. Inspired by this, Schwarz et al. [37] prepared some **GA** glycoside structural analogues **60**–**66** (Scheme 5) utilizing methyl glycyrrhetinate (compound **1**, Scheme 1) as starting material.

Their antitumor activity was evaluated in a SRB assay on various tumor cell lines. These derivatizations did not result in increased cytotoxicity, with the exception of compound **64** which showed IC_50_ values as low as 9.48 µM on breast carcinoma MCF-7 cells, which was twice the activity of **GA**–**Me**. It seemed that there was no correlation between the monosaccharide structure and the cytotoxicity, and similar results could also be found in [36,38,39]. The cytotoxicity (IC_50_ values in µM) of **60**–**66** in a panel of various cancer cell lines is summarized in Table 5.

**Scheme 6:**
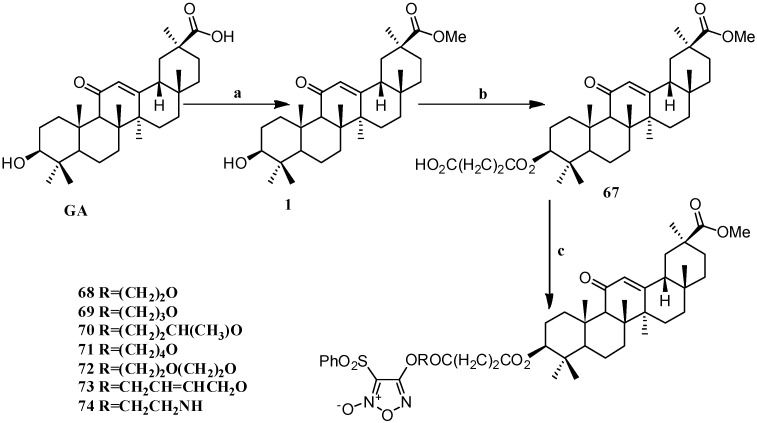
Synthesis of the **GA** furan-based nitric oxide (NO)-releasing derivatives **67**–**74**. *Reagents and conditions*: (a) CH_3_OH, *p*-TSA; (b) succinic anhydride, DMAP, dry DCM, 15 h; (c) phenylsulfonyl furans, DCC, DMAP, dry DCM, 24 h.

Lai et al. [40] designed and synthesized a series of novel furan-based nitric oxide (NO)-releasing derivatives of **GA 68**–**74** (Scheme 6) as antitumor agents. According to the MTT assay results, compounds **68**–**74** displayed increased anti-HCC (HepG2, BEL-7402) activity (IC_50_ 2.90–36.52 µM on HepG2, IC_50_ 2.94–19.92 µM on BEL-7402) compared with **GA** (IC_50_ > 50 µM on HepG2, BEL-7402). The most active compound was **74**, showing IC_50_ values as low as 2.90 µM, 2.94 µM on HepG2 and BEL-7402, respectively. These findings might provide more information for the design of new chemotherapeutic reagents for the intervention on human HCC in the clinic. The cytotoxicity (IC_50_ values in µM) of **68**–**74** in a panel of various cancer cell lines is summarized in Table 6.

**Figure 2:**
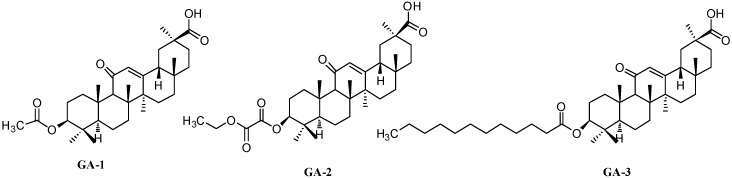
Structures of **GA-1**, **GA-2** and **GA-3**.

After forming long chains with ester bonds at C-3, Kumar Yadav et al. [41] found the **GA-1**, **GA-2** and **GA-3** (Figure 2) expressed significant antitumor activity against the human lung cancer cell line A-549 with pred. log IC_50_ = 1.182, 1.044, 1.274 µM according to the quantitative structure-activity relationship (QSAR) model. The cytotoxicity (IC_50_ values in µM) of **GA-1**, **GA-2** and **GA-3** on A-549 is summarized in Table 7.

#### 2.1.2. Structural Modifications at the Skeleton of Ring-A

**Figure 3:**
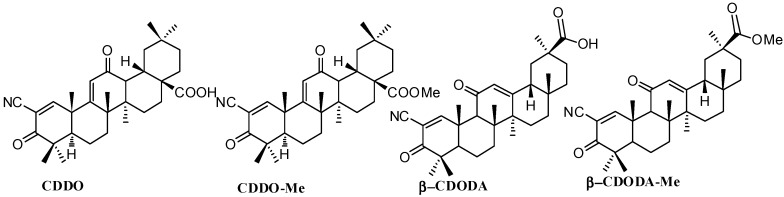
Structures of **CDDO**, **CDDO-Me**, **β-CDODA** and **β-CDODA-Me**.

**Scheme 7:**
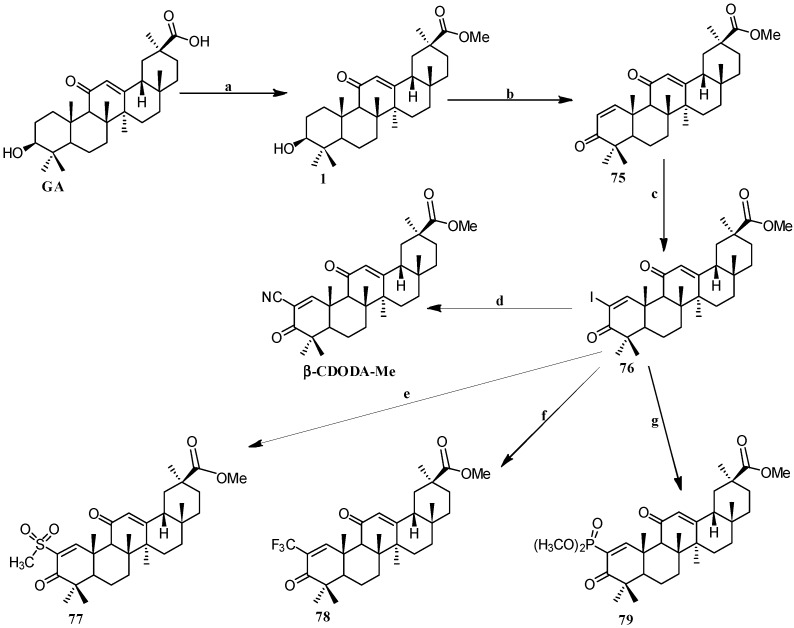
Synthesis of the **GA** 2-substituted derivatives **75**–**79**. *Reagents and conditions*: (a) CH_2_N_2_, Et_2_O, 0 °C; (b) IBX, DMSO, 21 h, 80–85 °C; (c) iodine, pyridine, tetrahydrofuran; (d) CuCN, NMP, 2 h, 130 °C; (e) CH_3_SO_2_Na, CuI, DMSO, 20 h, 120–125 °C; (f) CuI, methyl-2,2-difluoro-2-(fluorosulfonyl) acetate, DMF/HMPT, 20 h, 70 °C; (g) dimethyl phosphite, Cs_2_CO_3_, *N*,*N*-dimethylethylenediamine, toluene, 26 h, 95–100 °C.

Previous studies revealed that some triterpenoid derivatives which contained a 2-cyano-1-en-3-one functionality on ring-A, such as the oleanoic acid derivatives **CDDO** (Figure 3) and its methyl ester **CDDO-Me** (Figure 3), exerted potent cytotoxic activity in various cancer cell lines [42,43]. Similar results were also obtained with **GA** and betulinic acid derivatives containing a 2-cyano-1-en-3-one function, for example **β-CDODA-Me** [44,45] (Figure 3). Inspired by this, Chadalapaka et al. [31] synthesized some **β-CDODA-Me** analogs **75**–**79** (Scheme 7) with different electronegative 2-substituents including iodo, cyano, trifluoromethyl, dimethylphosphonyl and methanesulfonyl groups. The cell culture studies showed that the anti-proliferative activity of methyl derivative (**β-CDODA-Me**) on bladder and pancreatic cancer cells was more potent than that of the free acid (**β-CDODA**). This was consistent with a previous report [46]. Among the derivatives, 2-cyano and 2-trifluoromethyl ones showed the highest anti-proliferation activity. However, compound **79** and compound **77** were relatively inactive, showing higher IC_50_ values ranging from 3.34 to 11.97 µM than the corresponding 2-cyano and 2-trifluoromethyl derivatives on the four cell lines. It could be seen that their relative potencies were dependent on the cell context: 2-trifluoromethyl derivative (compound **78**) (IC_50_ 0.38 µM in KU7, IC_50_ 0.82 µM in Panc-1, IC_50_ 1.14 µM in Panc-28) was more active than **β-CDODA-Me** (IC_50_ 1.59 µM in KU7, IC_50_ 1.22 µM in Panc-1, IC_50_ 1.80 µM in Panc-28), whereas **β-CDODA-Me** was more active in 253JB-V cells, showing IC_50_ values as low as 0.25 µM, lower than that of the compound **78 (**IC_50_ 0.67 µM). The results provided a new way for the structural modifications of **GA**. The cytotoxicity (IC_50_ values in μM) of **76**–**79** in a panel of various cancer cell lines is summarized in Table 8.

**Scheme 8:**
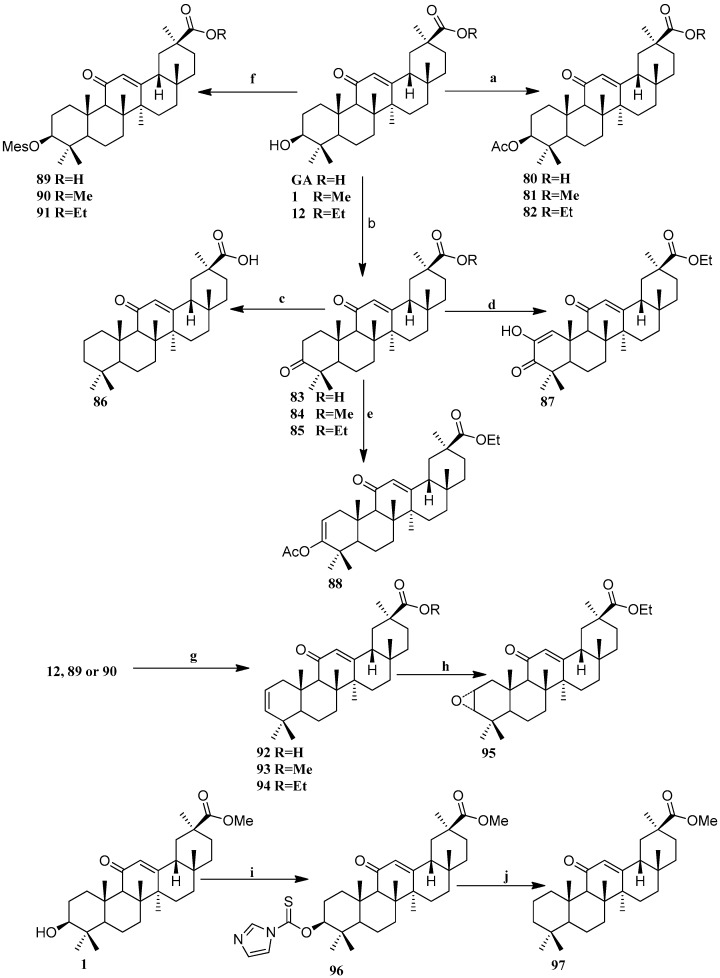
Synthesis of the C-2 and C-3 modified **GA** derivatives **80**–**97**. *Reagents and conditions*: (a) AcCl, pyridine, CH_2_Cl_2_, 2 h, 25 °C; (b) Jones reagent, 20–60 min, 25 °C; (c) KOH, hydrazine, ethylene glycol, 24 h, 200 °C; (d) periodic acid, DMSO, 3 days, −50 °C; (e) HOAc, p-TsOH, 24 h, 80 °C; (f) MeSO_2_Cl, pyridine (or Et_3_N for 15), 1–70 h, 25 °C; (g) for 92: K_2_CO_3_, DMF, 24 h, 120 °C; for 93: Bu_4_NF, DMF, 4 days, 102 °C; for 94: PPh3, 3,3-dimethylglutarimide, DEAD, THF, 24 h, 25 °C; (h) *m*-CPBA, CH_2_Cl_2_, 20 h, 25 °C; (i) 1,1′-thiocarbonyldiimidazole, 1,2-dichloroethane, 70 h, 100 °C; (j) Bu_3_SnH, AIBN (cat.), toluene, 40 h, 115 °C.

In order to alter the lipophilicity of **GA**, several functional modifications were carried out at the C-2 and/or C-3 positions in ring-A by Csuk et al. [46] and a series of derivatives **80**–**97** (Scheme 8) were obtained. Their cytotoxicity was investigated on eight different human tumor cell lines. According to the SRB assays, most of the derivatives showed lower antitumor activity than **GA**. Acetylated **GA** derivatives **80**–**82** and oxidized **GA** derivatives **83**–**85** did not show any significant antitumor activity. Deoxidized **GA** derivatives **86** and **97** were relatively active, showing IC_50_ < 20 µM in several tested cancer cell lines. The cytotoxicity (IC_50_ values in μM) of **80**–**95**, **97** in a panel of various cancer cell lines is summarized in Table 9.

**Scheme 9:**
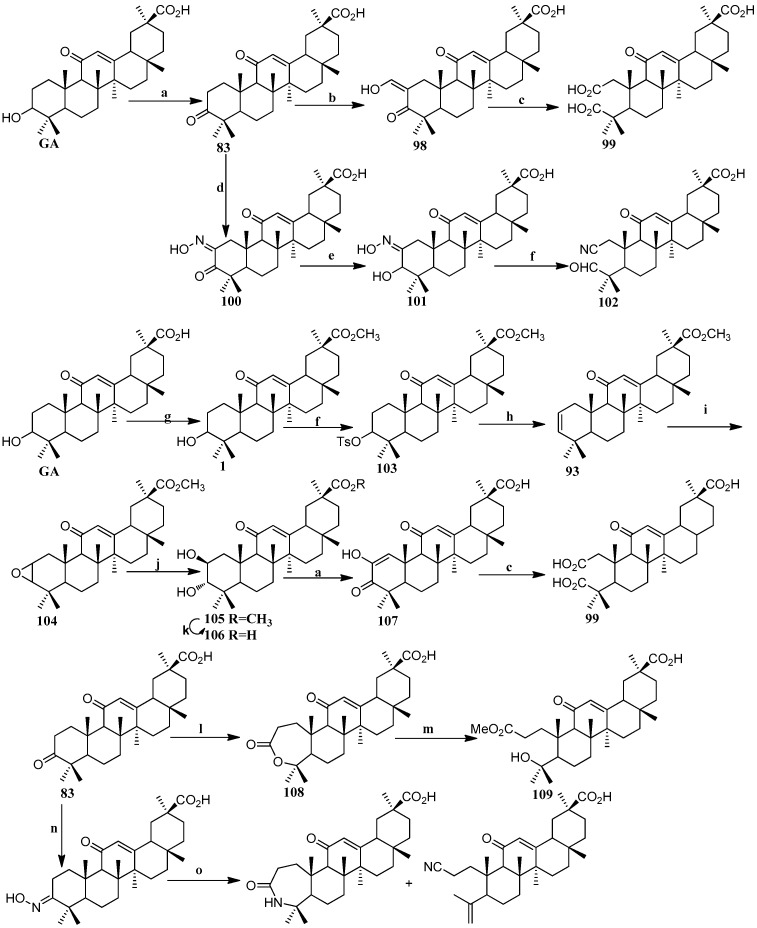
Synthesis of ring A modified **GA** derivatives **98**–**112**. *Reagents and conditions*: (a) Jones’ reagent; (b) HCO_2_Et, NaOMe; (c) NaOMe, H_2_O_2_; (d) *t*-BuOK/*t*-BuOH, *n*-BuONO; (e) NaBH_4_; (f) *p*-TsCl; (g) CH_3_I, K_2_CO_3_; (h) LiBr, Li_2_CO_3_; (i) *m*-CPBA, K_2_CO_3_; (j) HClO_4_; (k) KOH; (l) *m*-CPBA, NaHCO_3_; (m) NaOMe; (n) NH_2_OH·HCl; (o) p-TsCl, DMAP.

In the search of new **GA** derivatives as antitumor agents, Jun et al. [47] employed **GA** as precursor and synthesized a series of **GA** derivatives **98**–**112** (Scheme 9) with major changes to ring-A. The preliminary pharmacological study showed compound **98**, **100**, **101**, **105**, **106**, **110** with hydroxyl groups displayed some cytotoxicity on HepG-2. The derivative **105** with two hydroxyl groups at C-2 and C-3 displayed more potent activity than **GA** showing IC_50_ as low as 0.22 µM on HepG-2.

It seemed that the number and location of hydroxyl groups in ring-A had an important influence on the antitumor activity of **GA** derivatives. The cytotoxicity (IC_50_ values in μM) of **98**–**112** on HepG-2 os summarized in Table 10.

### 2.2. Structural Modifications on Ring-C

**Scheme 10:**
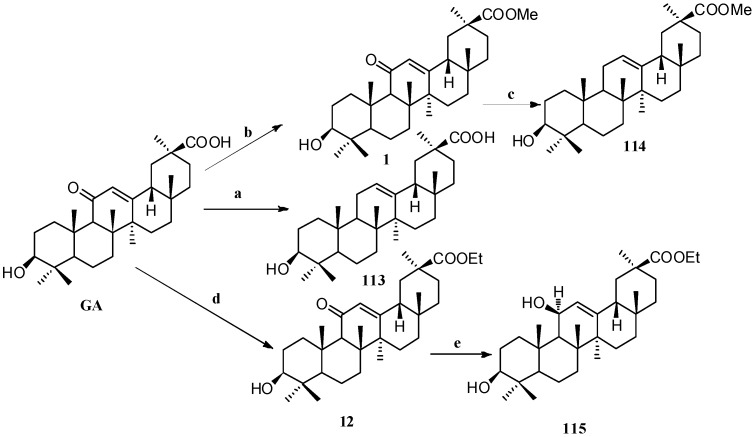
Synthesis of ring C modified **GA** derivatives **113**–**115**. *Reagents and conditions*: (a) Zinc dust, conc. HCl, dioxane, 25 °C, 24 h; (b) MeI, K_2_CO_3_, DMF, 25 °C, 24 h; (c) BH_3_-THF, THF, citric acid, 25 °C, 20 h; (d) EtI, K_2_CO_3_, DMF, 25 °C, 24 h; (e) BH_3_-THF, THF, Na_2_CO_3_, 25 °C, 4 days.

The studies on structural modifications at ring-C were mainly focused on the carbonyl function located at C-11. According to Fiore and Salvi [48,49], a ketone group at position C-11 was the primary cause for the apoptotic activity of **GA** derivatives, but the research conducted by Csuk et al. [50] showed that there was no direct relation between the presence of the C-11 ketone group and the apoptotic activity of the compounds. Also, esterification at C-30 was important, as mentioned above. Six compounds (Scheme 10) were tested in a SRB assay for cytotoxicity screening on 12 tumor cell lines and mouse embryonic fibroblasts (NIH3T3) which showed that **GA** and compound **113** nearly had the same activity on tumor cells, but after esterification at C-30, compounds **1** and **114** showed a relatively high cytotoxicity against the tested tumor cell lines. For the fibroblasts and most of the tumor cell lines, the toxicity of compound **114** was reduced, while the cytotoxic effect on the tumor cells of compounds **12** and **115** was similar to their effect on NIH3T3 cells. However, according to Lin et al. [51], when **GA** was converted into **11-DOGA**, it showed higher toxicity toward gastric cancer cells both in vivo and in vitro, so the relation between the existence of the C-11 ketone group and the apoptotic activity should be further studied. The cytotoxicity (IC_50_ values in μM) of **1**, **12**, **113**–**115** in a panel of various cancer cell lines is summarized in Table 11.

### 2.3. Structural Modifications on Ring-E

**Scheme 11:**
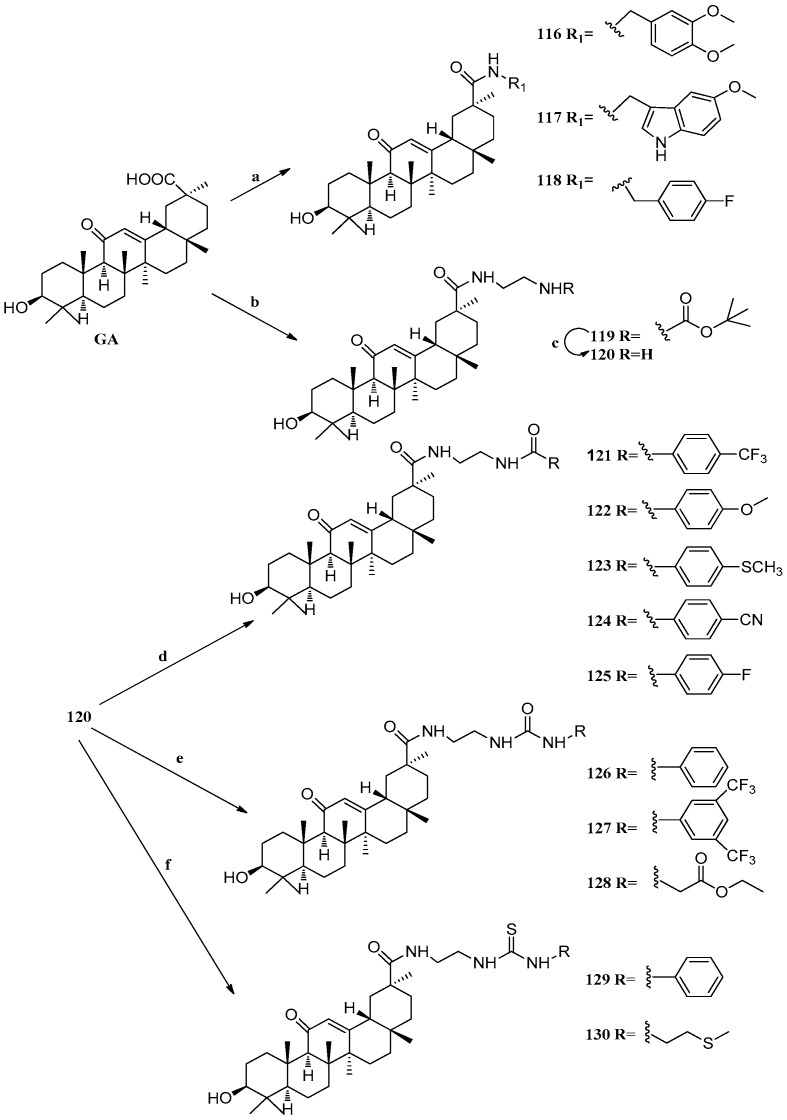
Synthesis of ring E modified **GA** derivatives **116**–**130**. *Reagents and conditions*: (a) 1. DCC, HOBt, DIPEA, DMF, r.t., 30 min; 2. R_1_NH_2_, r.t., overnight; (b) 1. DCC, HOBt, DIPEA, DMF, r.t., 30 min; 2. H_2_N(CH_2_)_2_NHBoc, r.t., overnight; (c) TFA, DCM, 0 °C, 3 h; (d) DMAP, RCOCl, DCM; (e) THF, RNCO, r.t., 20 h; (f) THF, RNCS, r.t., 20 h; (g) Jones reagent, acetone, 0 °C, 45 min.

The C-30 position in **GA** has been widely exploited and hundreds of derivatives have been reported in the literature. To increase the antitumor activity of **GA** and to obtain potent cytostatic compounds, Lallemand et al. [52] synthesized a series of **GA** amide derivatives **116**–**130** (Scheme 11) by coupling **GA** with various amines. The antitumor activity screening showed that compound **127** appeared to be the most potent one, with single-digit micro molarity IC_50_ values in a panel of eight cancer cell lines. Further pharmacokinetic studies by the same group suggested that compound **127** was rapidly distributed (t_1/2_dist of ~3 min) but slowly eliminated (t_1/2_elim = ~77 min). This study was helpful in producing this kind of **GA** antitumor derivatives.

Similarly, Shi et al. [53] synthesized biotinylated **GA** (**BGA**) by introducing biotin into the C-30 carboxyl of **GA**, and evaluated its antitumor effects on mouse B16 melanoma cells and BEL 7402 cells. The result showed that the biotin group in **BGA** had no influence on the antitumor effects of **GA**. The cytotoxicity (IC_50_ values in μM) of **116**–**130** in a panel of various cancer cell lines is summarized in Table 12.

**Scheme 12:**
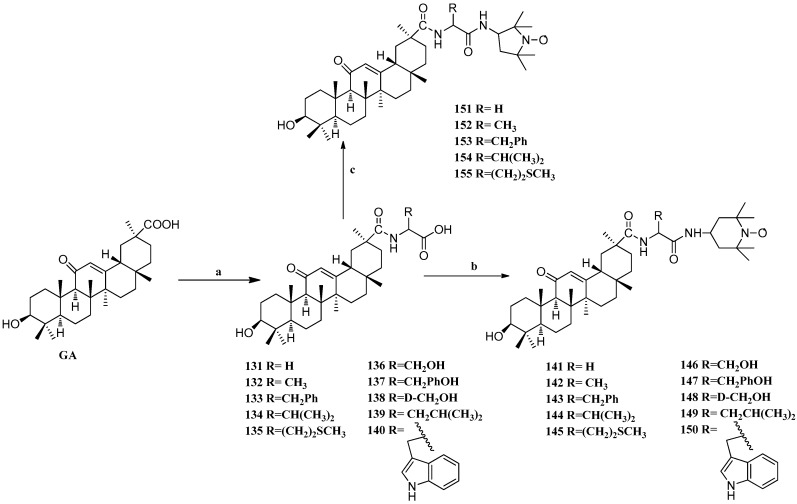
Synthesis of ring E modified **GA** derivatives **131**–**155**. *Reagents and conditions*: (a) (i) amino acid methyl ester EDCI/HOBt/Et_3_N, DMF; (ii) 4N NaOH THF/MeOH; (b) EDCI/HOBt/Et_3_N DMF, r.t., overnight; (c) EDCI/HOBt/Et_3_N DMF, r.t., overnight.

Guided by previous results indicating that incorporation of a stable nitroxyl radical or amino acids into antitumor molecules could increase their activity and decrease their toxicity [34,54,55], Liu et al. [56] designed and synthesized a series of **GA** derivatives **131**–**140** (Scheme 12) by introducing a nitroxyl functionality and amino acid segments into **GA**.

The in vitro cytotoxicity screening showed that compounds **131**–**140** with only various free amino acids at C-30 showed no significant cytotoxicity (GI_50_ > 70 µM). However, incorporation of a piperidine (compounds **141**–**150**) or pyrroline (compounds **151**–**155**) nitroxyl radical at the terminus of the C-30 side chains could significantly enhance the cytotoxic effects. Among the new derivatives, compound **150** with a tryptophan amino moiety and a piperidine nitroxyl radical showed the greatest cytotoxicity (GI_50_ 13.7–15.0 µM), five-fold more potent than **GA**. These results suggested that the incorporation of a nitroxyl functionality and amino acid segments into the C-30 carboxyl group of **GA** might contribute to improve its cytotoxicity. The cytotoxicity (GI_50_ values in μM) of **141**–**155** in a panel of various cancer cell lines is summarized in Table 13.

Inspired by previous studies indicating that esterification of glycyrrhetinic acid (**GA**) with dehydrozingerone (**DZ**) resulted in a novel cytotoxic **GA**–**DZ** conjugate, Tatsuzaki et al. [57] synthesized a series of triterpenoid—dehydrozingerone derivatives by combining **DZ** analogs with different triterpenoids, such as oleanoic acid (**OA**), ursolic acid (**UA**), glycyrrhetinic acid (**GA**).

**Scheme 13:**
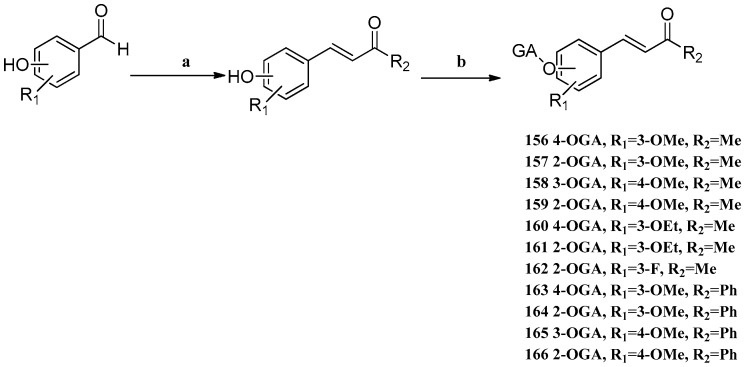
Syntheses of GA–DZ derivatives **156**–**166**. *Reagents and conditions*: (a) 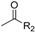, 1N NaOH (for R_2_ = Me), 5N KOH (for R_2_ = Ph); (b) GA, EDCI, DMAP, CH_2_Cl_2_.

The in vitro antitumor assay indicated that most of the **GA**–**DZ** conjugates **156**–**166** (Scheme 13) showed significant antitumor activity. In particular, compounds **156**–**158** exhibited prominent cytotoxicity against LN-Cap, 1A9, and KB cells with ED_50_ values of 0.6, 0.8 and 0.9 µM. However, similar conjugates between **DZ** and **OA** or **UA** were inactive suggesting that the **GA** component was critical for activity. The cytotoxicity (ED_50_ values in μM) of **156**–**166** in a panel of various cancer cell lines is summarized in Table 14.

**Scheme 14:**
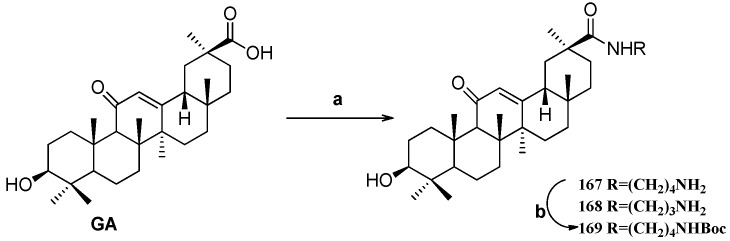
Synthesis of the **GA** amide derivatives **167**–**169**. *Reagents and conditions*: (a) K_2_CO_3_, diamine, DMF, 25 °C, 20 h; (b) Boc_2_O, Et_3_N, MeOH, 25 °C, 20 h.

**Scheme 15:**
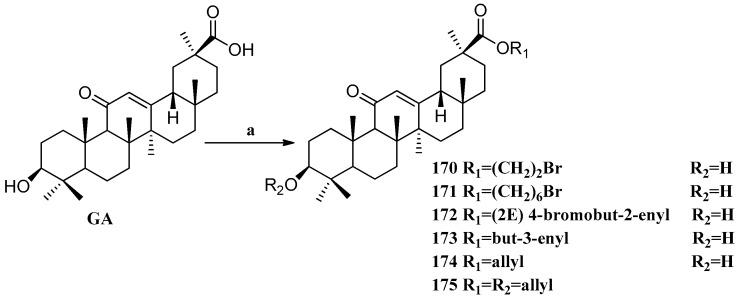
Synthesis of the **GA** ester derivatives **170**–**175**. *Reagents and conditions*: (a) K_2_CO_3_, alkyl halide, DMF, 25 °C, 20 h.

In the search of new **GA** derivatives as antitumor agents, Csuk et al. [58] performed some variations at C-30 of **GA**, including esterification, the formation of amides and a nitrile. The antitumor evaluation showed the amide derivatives like compounds **167**–**169** (Scheme 14) showed no cytotoxic activity at 30 µM concentration, but nearly all the ester derivatives like compounds **170**, **172**–**174** (Scheme 15) exhibited high cytotoxic activity. In particular, compound **172** exhibited potent cytotoxic activity on SW1736 cells (IC_50_ = 1.88 µM), while compound **175** esterified at C-30 and etherified at C-3 almost showed no cytotoxic activity (IC_50_ > 30 µM) against seven tested human tumor cell lines. This suggested that not only the type of the chemical bonding but also the position of substituent groups affects the antitumor activity. This study greatly enriched the modification strategy of the carbonyl group. The cytotoxicity (IC_50_ values in μM) of **167**–**175** in a panel of various cancer cell lines is summarized in Table 15.

### 2.4. Structural Modifications of Multiple Rings

In an attempt to improve the pharmacological activity of **GA**, structural modification at multiple rings has been reported. Structural modifications of multiple rings in **GA** has focused on the A, C, and E rings, especially at A and E ring. Shen et al. [59,60] reported syntheses and antitumor activity of some **GA** derivatives by simultaneously modifying the C-3 hydroxyl group and the C-30 carboxyl group in **GA**. They found when the carbon chain of the linking group was 2 to 4, the activity increased as the carbon chain was lengthened, while when the carbon chain length of the linking group was 5, the activity decreased. Meanwhile, they also found that when there were nitrate moieties at C-3 and C-30 simultaneously; the antitumor activity of the compounds was enhanced.

**Scheme 16:**
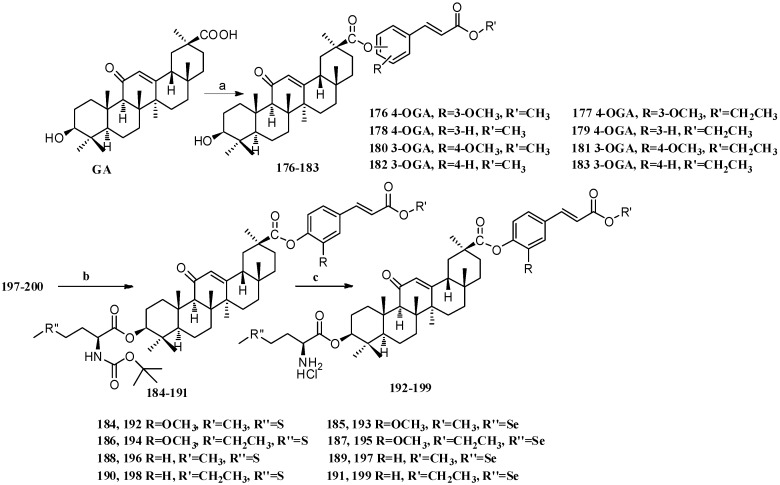
Synthesis of multiple rings modified **GA** derivatives **176**–**199**. *Reagents and conditions*: (a) ferulic acid analogs, EDCI, DMAP, CH_2_Cl_2_, r.t.; (b) Boc-L-methionine or Boc-L-selenomethionine, EDCI, DMAP, CH_2_Cl_2_, r.t.; (c) HCl (gas) in CH_2_Cl_2_, r.t.

Starting from **GA**, Li et al. [61] synthesized a series of **GA** derivatives **176**–**199** (Scheme 16) in which the 30-carboxyl group was modificated by ferulic acid analogs and the 3-hydroxyl group was coupled with amino acids. The MTT assay results showed that most of the derivatives exhibited much higher antitumor activity than **GA** against cancer cell lines (MCF-7 cells, MDA-MB-231) and lower cytotoxicity against normal cells (hTERT-RPE1 cells).

Among the derivatives, compound **193** was the most active one (IC_50_ 1.88 + 0.20 µM for MCF-7; IC_50_ 1.37 + 0.18 µM for MDA-MB-231). The results displayed that introduction of a lipophilic fragment or amino acid groups into C-3 and C-30 might increase the antitumor activity. The cytotoxicity (IC_50_ values in μM) of **176**–**199** in a panel of various cancer cell lines is summarized in Table 16.

In order to further improve the antitumor activity of **GA**, Song et al. [62] designed and synthesized a series of novel **GA** derivatives by modifying the structure at the C-3 hydroxyl or C-11 carbonyl or C-30 carboxyl.

**Scheme 17:**
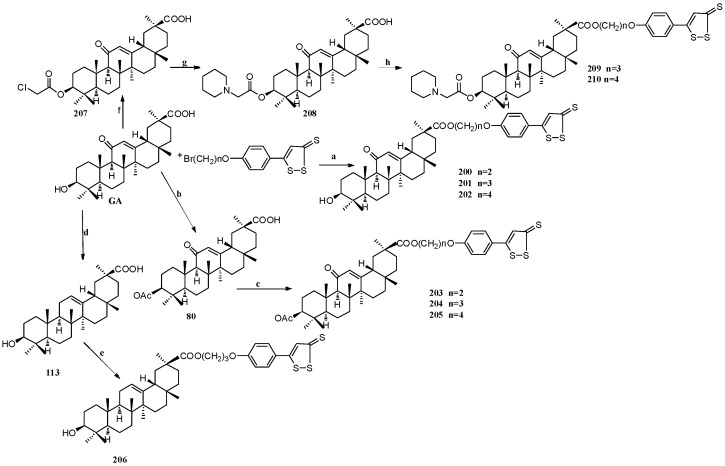
Synthesis of multiple rings modified **GA** derivatives **200**–**210**: *Reagents and conditions*: (a) K_2_CO_3_, cat. KI, 60 °C, 12 h, chromatography; (b) Ac_2_O, Py, r.t., 3 h, chromatography; (c) K_2_CO_3_, cat. KI, 224, or 225 or 226, 60 °C, 12 h; (d) Zn (containing 10% HgCl_2_), concentrated. HCl, 1,4-dioxane, 20 °C, 2 h, chromatography; (e) K_2_CO_3_, cat. KI, 227, 60 °C, 12 h, chromatography; (f) ClCH_2_COCl, Py, THF, r.t., 4 h; (g) Et_3_N, THF, refluxing, 10 h; (h) K_2_CO_3_, cat. KI 230 or 231 60 °C, 12 h, chromatography.

The biological activity evaluation showed that compound **203** (Scheme 17) exhibited the most promising antitumor activity against tumor cell lines MDA-MB-231 cells, DU-145 cells and Hep-G2 cells (IC_50_ 10.01 µM for HepG2, 11.96 µM for DU-145 and 17.8 µM for MDA-MB-231), which was much better than starting material **GA** (IC_50_ values of 74.35, 69.40, 72.65 µM, respectively). What’s more, compound **200** with linker *n* = 2 and compound **205** with linker *n* = 4 also showed higher antitumor activity than **GA** on all tested tumor cell lines. But other compound, such as **201**, **202**, **204**, showed weak anti-proliferative effect due to their poor solubility. The cytotoxicity (IC_50_ values in μM) of **200**–**206**, **209**, **210** in a panel of various cancer cell lines is summarized in Table 17.

## 3. Conclusions

Glycyrrhetinic Acid was found to possess remarkable anti-proliferative and apoptosis-inducing activity against various cancer cell lines. A number of structural modifications of **GA** were carried out to synthesize new potential antitumor agents. As for the many synthetic strategies reported in this review, they can be summarized as follows: (i) introduction of aminoalkyl, amino acid, sugar and other groups into the hydroxyl group at C-3 by esterification; (ii) oxidation or elimination of the hydroxyl group at C-3, introduction of functional groups at C-2, opening or increasing the number of atoms of ring-A; (iii) elimination of the C-11 ketone group in ring-C; (iv) esterification or amidation of the carboxyl group at C-30 in ring-E; (v) esterification at the C-3 hydroxyl group and C-30 carboxyl group simultaneously, elimination of the ketone group at C-11 and esterification at C-30 simultaneously.

**Figure 4:**
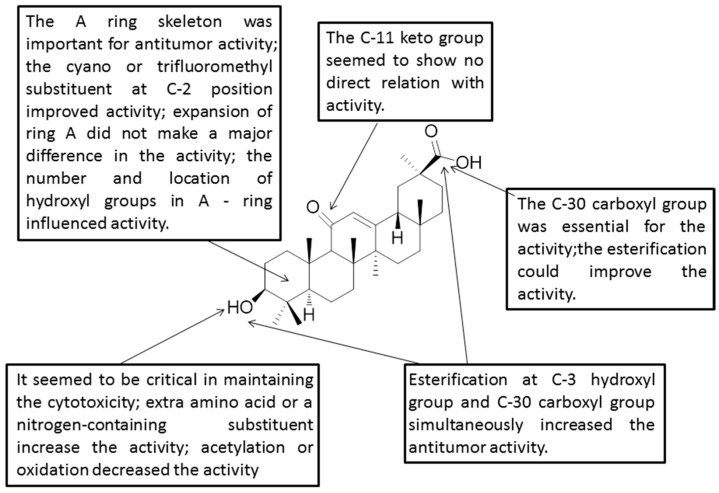
Structure-activity relationships of GA.

To some extent, the reported **GA** derivatives and their biological activity confirmed that there are many factors affecting the antitumor activity, such as the kind, quantity and position of substituents, and the type of chemical bonding. The published studies of **GA** derivatives as the antitumor agents have provided us much useful information which was as follows and is summarized in Figure 4:

1. The hydroxyl at the C-3 position seems to be critical in maintaining the cytotoxicity. The introduction of an extra amino acid or a nitrogen-containing substituent was found to be beneficial to increase the cytotoxicity, but the acetylation or oxidation of the hydroxyl group at the C-3 position resulted in a decreased anti-proliferative activity.
2. The A ring skeleton plays an important role in eliciting antitumor activity. A cyano or trifluoromethyl substituent at C-2 position of **GA** improved the cytotoxicity. Expansion of ring A did not make a major difference in the cytotoxicity, but the number and location of hydroxyl groups in the A-ring has an important influence on the antitumor activity.
3. The C-11 keto group of C ring seems to show no direct relation with cytotoxicity.
4. The C-30 carboxyl group is essential for cytotoxicity. Esterification at the C-30 carboxylic acid could improve the antitumor efficacy.
5. Esterification at the C-3 hydroxyl group and C-30 carboxyl group simultaneously increased the antitumor activity.

The chemical methods for the structural modifications of **GA** are efficient but the strategies were long and complicated and often involve harsh reaction conditions, therefore, in the future studies structure-activity relationships should be a prerequisite and focused on obtaining highly effective and low-toxicity antitumor derivatives of **GA**.

## Figures and Tables

**Table 1:** Cytotoxicity (IC_50_ values in µM) of **1**–**11** in a panel of various cancer cell lines.

Cell Lines	GA	1	2	3	4	5	6	7	8	9	10	11
518A2	83.92	27.54	25.43	5.24	3.79	2.55	2.02	1.09	1.27	3.49	3.12	4.33
8505C	86.50	26.07	26.08	15.86	3.37	2.12	1.78	1.68	2.13	3.35	6.18	7.60
A253	80.78	19.42	25.54	6.19	3.64	2.56	2.27	1.12	1.74	3.01	4.65	5.48
A2780	74.57	25.54	23.77	6.01	4.39	2.43	2.00	1.36	1.14	2.80	3.30	3.63
A549	82.76	23.50	24.80	8.39	5.15	3.31	2.52	1.59	2.21	4.08	2.23	5.16
DLD-1	81.21	26.12	17.36	6.13	4.39	2.66	2.40	0.91	1.25	3.96	4.50	5.53
FADU	84.55	23.41	23.56	12.44	5.57	3.51	3.30	1.78	2.20	4.26	5.54	5.65
HCT-11	78.83	22.10	14.41	5.13	4.30	2.41	2.19	1.17	1.70	3.53	3.44	3.86
HCT-8	78.85	24.36	13.39	3.97	2.37	1.51	1.38	0.62	0.89	2.92	2.42	4.07
HT-29	80.09	27.54	16.91	5.34	2.90	1.69	1.28	0.59	0.86	2.76	2.06	2.73
LIPO	81.44	20.47	25.39	14.55	3.89	2.57	1.93	1.59	1.44	4.36	5.48	6.93
MCF-7	84.70	22.14	25.22	6.69	3.55	2.45	1.79	1.17	0.98	3.89	3.33	2.68
SW1736	76.93	34.87	16.42	3.14	6.05	3.30	2.69	1.61	2.24	4.09	3.30	3.73
SW480	86.80	16.08	25.91	8.92	3.68	2.54	1.91	2.25	2.24	3.93	5.74	4.73

**Table 2:** Cytotoxicity (IC_50_ values in µM) of **12**–**32** in a panel of various cancer cell lines.

Compound	8505C	A253	A2780	A549	DLD-1	LIPO	Average
**GA**	86.50 ± 4.20	80.78 ± 4.04	74.57 ± 3.73	82.76 ± 4.14	81.21 ± 4.06	81.44 ± 4.07	81.4 ± 4.07
**12**	24.58 ± 1.23	25.04 ± 1.25	26.96 ± 1.35	22.74 ± 1.14	28.14 ± 1.41	27.66 ± 1.38	24.39 ± 1.22
**13**	14.24 ± 0.71	15.76 ± 0.79	24.95 ± 1.25	14.41 ± 0.72	27.61 ± 1.38	15.93 ± 0.80	19.21 ± 0.96
**14**	8.10 ± 0.41	10.67 ± 0.54	20.32 ± 1.18	6.15 ± 0.31	22.69 ± 1.13	11.54 ± 0.80	13.76 ± 0.69
**15**	>30	>30	>30	>30	>30	>30	>30
**16**	>30	>30	>30	>30	>30	>30	>30
**17**	>30	>30	>30	>30	>30	>30	>30
**18**	7.45 ± 0.37	6.26 ± 0.31	5.99 ± 0.30	6.42 ± 0.32	8.59 ± 0.43	7.54 ± 0.38	7.04 ± 0.35
**19**	4.31 ± 0.22	3.61 ± 0.18	2.98 ± 0.15	2.77 ± 0.14	4.49 ± 0.22	4.30 ± 0.22	3.74 ± 0.19
**20**	2.55 ± 0.13	2.50 ± 0.13	1.72 ± 0.09	2.40 ± 0.12	2.51 ± 0.13	2.52 ± 0.13	2.37 ± 0.12
**21**	5.32 ± 0.27	3.59 ± 0.18	3.90 ± 0.20	5.39 ± 0.27	5.61 ± 0.28	4.32 ± 0.22	4.69 ± 0.23
**22**	3.87 ± 0.19	2.33 ± 0.12	2.59 ± 0.13	3.43 ± 0.17	3.72 ± 0.19	2.74 ± 0.14	3.11 ± 0.16
**23**	2.32 ± 0.12	2.23 ± 0.11	1.77 ± 0.09	2.18 ± 0.11	2.74 ± 0.14	2.38 ± 0.12	2.27 ± 0.11
**24**	2.76 ± 0.14	2.01 ± 0.10	2.24 ± 0.11	2.65 ± 0.13	2.54 ± 0.13	2.74 ± 0.14	2.49 ± 0.12
**25**	3.49 ± 0.17	3.51 ± 0.18	2.08 ± 0.10	3.43 ± 0.17	5.54 ± 0.28	3.53 ± 0.18	3.60 ± 0.18
**26**	1.96 ± 0.10	2.68 ± 0.13	1.31 ± 0.07	1.78 ± 0.09	3.52 ± 0.18	3.49 ± 0.17	2.46 ± 0.12
**27**	4.79 ± 0.24	5.03 ± 0.25	3.54 ± 0.18	5.07 ± 0.25	4.54 ± 0.23	4.81 ± 0.24	4.63 ± 0.23
**28**	3.10 ± 0.16	3.49 ± 0.17	2.85 ± 0.14	3.51 ± 0.18	5.02 ± 0.25	3.57 ± 0.18	3.59 ± 0.18
**29**	3.19 ± 0.16	3.05 ± 0.15	1.73 ± 0.09	2.76 ± 0.14	4.54 ± 0.23	3.25 ± 0.16	3.09 ± 0.15
**30**	>30	>30	>30	>30	>30	>30	>30
**31**	>30	>30	>30	>30	>30	>30	>30
**32**	>30	>30	>30	>30	>30	>30	>30

**Table 3:** Cytotoxicity (IC_50_ values in µM) of **33**–**40**, **43**, **44** in a panel of various cancer cell lines.

Cell Lines	35	36	37	38	39	40	43	44
518A2	10.90 ± 0.55	1.75 ± 0.09	17.19 ± 0.86	17.94 ± 0.90	>100	>100	39.24 ± 1.96	47.72 ± 2.39
8505C	12.97 ± 0.45	1.76 ± 0.09	15.82 ± 0.79	17.00 ± 0.85	>100	>100	45.36 ± 2.27	61.57 ± 3.08
A253	7.99 ± 0.40	1.28 ± 0.06	15.07 ± 0.75	13.80 ± 0.69	>100	>100	30.47 ± 1.52	53.07 ± 2.65
A2780	8.84 ± 0.44	1.65 ± 0.08	17.29 ± 0.86	18.24 ± 0.91	>100	>100	22.44 ± 1.12	29.19 ± 1.46
A549	10.94 ± 0.55	1.77 ± 0.09	19.82 ± 0.99	21.20 ± 1.06	>100	>100	31.59 ± 1.58	60.96 ± 3.05
Lipo	11.35 ± 0.57	1.74 ± 0.09	16.67 ± 0.83	18.78 ± 0.94	>100	>100	40.62 ± 2.03	54.77 ± 2.74
MCF-7	7.35 ± 0.36	1.27 ± 0.06	17.47 ± 0.87	16.96 ± 0.85	>100	>100	16.89 ± 0.84	29.26 ± 1.46
SW1736	16.68 ± 0.83	2.33 ± 0.12	17.13 ± 0.86	19.24 ± 0.96	>100	>100	20.85 ± 1.04	38.50 ± 1.93
Average	10.88 ± 0.54	1.69 ± 0.08	17.06 ± 0.85	17.90 ± 0.90	>100	>100	30.93 ± 1.55	46.77 ± 2.34
NiH3T3	14.74 ± 0.74	39.09 ± 1.95	23.09 ± 1.15	24.42 ± 1.22	>100	>100	16.89 ± 0.84	33.63 ± 1.68
F	1.35	23.13	1.35	1.36			0.55	0.72

**Table 4:** Cytotoxicity (IC_50_ values in µM) of **45**–**59** in a panel of various cancer cell lines.

Compound	8505C	A253	A2780	A549	DLD-1	LIPO	MCF-7
**45**	2.92	2.26	2.24	2.26	3.35	3.56	2.25
**46**	2.50	2.46	1.83	2.13	3.42	2.50	2.49
**47**	9.62	5.56	4.58	6.91	11.64	7.96	5.49
**48**	16.93	6.41	5.50	9.94	8.70	16.15	4.60
**49**	11.47	7.48	12.56	14.48	12.45	22.32	6.06
**50**	>30	>30	6.89	>30	>30	>30	>30
**51**	>30	>30	>30	>30	>30	>30	>30
**52**	>30	>30	>30	>30	>30	>30	>30
**53**	3.47	3.41	2.13	3.39	3.41	3.54	2.73
**54**	3.52	3.52	2.48	3.38	4.49	4.54	3.40
**55**	5.48	4.05	4.94	5.43	6.27	5.95	4.03
**56**	4.02	3.76	4.06	3.88	4.38	4.02	2.46
**57**	2.89	4.04	2.59	2.35	1.48	0.80	3.01
**58**	2.49	2.21	1.98	2.53	3.01	2.70	1.55
**59**	2.40	2.43	1.58	2.43	2.27	2.51	1.75

**Table 5:** Cytotoxicity (IC_50_ values in µM) of **60**–**66** in a panel of various cancer cell lines (NA = not active).

Cell Lines	60	61	62	63	64	65	66	GA–Me
SW1736	NA	NA	NA.	23.87 ± 1.3	11.18 ± 0.9	21.38 ± 1.9	NA	34.87 ± 1.2
MCF-7	NA	16.7 ± 1.4	19.60 ± 1.4	NA	9.48 ± 1.4	20.11 ± 1.3	NA	22.14 ± 0.9
LIPO	NA	NA	NA	28.45 ± 2.1	NA	23.23 ± 1.3	NA	20.47 ± 1.1
DLD-1	NA	NA	NA	NA	NA	23.18 ± 1.7	NA	26.12 ± 1.0
A253	NA	NA	NA	27.25 ± 1.8	13.16 ± 0.9	19.70 ± 1.4	NA	19.42 ± 1.1
8505C	NA	NA	NA	NA	21.97 ± 0.6	22.77 ± 1.4	NA	26.07 ± 1.3
518A2	NA	NA	NA	28.92 ± 2.0	25.95 ± 0.8	23.26 ± 1.2	NA	27.54 ± 1.0
NiH3T3	NA	NA	NA	NA	NA	23.45 ± 0.1	NA	22.81 ± 0.6

**Table 6:** Cytotoxicity (IC_50_ values in µM) of **68**–**74** in a panel of various cancer cell lines.

Cell Lines	GA	68	69	70	71	72	73	74
HepG2	>50	18.18	13.41	26.03	36.52	15.67	7.90	2.90
BEL-7402	>50	7.85	9.22	6.03	8.20	19.92	7.37	2.94

**Table 7:** Cytotoxicity (IC_50_ values in μM) of **GA-1**, **GA-2** and **GA-3** in A-549.

Cell Lines	GA-1	GA-2	GA-3
A549	1.182	1.044	1.274

**Table 8:** Cytotoxicity (IC_50_ values in μM) of **76**–**79** and **β-CDODA-Me** in a panel of various cancer cell lines.

Compound	253JB-V	KU7	Panc-1	Panc-28
**76**	2.67	3.04	4.08	12.75
**77**	11.97	3.34	7.69	9.75
**78**	0.67	0.38	0.82	1.14
**79**	7.90	3.73	6.11	8.14
**β-CDODA-Me**	0.25	1.59	1.22	1.80

**Table 9:** Cytotoxicity (IC_50_ values in μM) of **80**–**95**, **97** in a panel of various cancer cell lines.

Compound	518A2	8505C	A2780	A549	DLD-1	LIPO	MCF-7	SW1736
**80–85**	>30	>30	>30	>30	>30	>30	>30	>30
**86**	18.33	19.28	28.83	>30	>30	28.74	21.87	16.56
**87**	29.82	27.69	14.84	26.62	29.56	24.80	28.68	27.00
**88**	>30	>30	>30	>30	>30	>30	>30	13.24
**89**	>30	29.42	>30	>30	>30	>30	>30	29.40
**90–92**	>30	>30	>30	>30	>30	>30	>30	>30
**93**	>30	>30	14.95	>30	>30	>30	>30	19.14
**94, 95**	>30	>30	>30	>30	>30	>30	>30	>30
**97**	23.69	24.30	10.39	>30	>30	25.52	>30	16.98

**Table 10:** Cytotoxicity (IC_50_ values in μM) of **98**–**112** in a panel of various cancer cell lines.

Cell Lines	98	99	100	101	102	103	104	105
HepG-2	61.70	>100	71.83	47.12	>100	>100	>100	0.22
	106	107	108	109	110	111	112	
HepG-2	59.98	>100	>100	>100	88.68	>100	>100	

**Table 11:** Cytotoxicity (IC_50_ values in μM) of **1**, **12**, **113**–**115** in a panel of various cancer cell lines.

Cell Lines	GA	113	1	114	12	115
518A2	83.92	71.49	27.54	34.54	25.23	51.52
8505C	86.50	78.52	26.07	33.88	24.58	52.80
A2780	74.57	62.78	25.54	23.58	26.96	57.01
A431	79.58	86.13	25.28	33.55	23.45	46.55
A549	82.76	79.13	23.50	31.59	22.74	48.97
DLD-1	81.21	90.50	26.12	31.73	28.14	52.80
HCT-116	78.83	87.70	22.10	31.82	21.58	47.78
HCT-8	78.85	88.76	24.36	31.34	43.42	44.32
HT-29	80.09	90.30	27.54	23.89	22.14	44.32
LIPO	81.44	73.88	20.47	34.81	27.66	52.80
MCF-7	84.70	90.19	22.14	34.37	18.61	48.97
SW1736	76.93	72.47	34.87	32.35	13.37	45.48
NIH3T3	18.52	68.70	22.81	42.22	23.66	43.16

**Table 12:** Cytotoxicity (IC_50_ values in μM) of **116**–**130** in a panel of various cancer cell lines.

Compound	A549	SKMEL	T98G	HS683	U373	PC3	MCF7	816F10
**GA**	>100	92	85	84	83	80	76	37
**116**	52	>100	91	59	43	34	34	37
**117**	40	>100	>100	57	75	43	38	31
**118**	33	82	46	56	42	33	31	32
**119**	43	60	73	63	57	41	37	48
**120**	31	>100	>100	58	32	31	59	30
**121**	47	49	62	38	55	53	28	36
**122**	63	42	77	58	75	72	46	31
**123**	37	38	54	36	37	47	30	31
**124**	68	35	77	67	76	72	27	31
**125**	28	37	35	31	29	30	25	28
**126**	29	49	30	28	30	32	28	31
**127**	7	9	12	6	6	8	4	4
**128**	29	65	71	42	42	46	42	41
**129**	31	38	25	8	29	9	30	34
**130**	38	33	35	36	35	39	30	33

**Table 13:** Cytotoxicity (GI_50_ values in μM) of **141**–**155** in a panel of various cancer cell lines.

Compound	A549	DU145	KB	Kbvin
**GA**	61.2 ± 2.33	64.9 ± 0.505	61.2 ± 0.118	62.3 ± 1.41
**141**	>70	>70	>70	>70
**142**	>70	>70	>70	>70
**143**	19.4 ± 0.909	19.3 ± 0.292	14.6 ± 0.448	14.9 ± 0.471
**144**	34.2 ± 1.88	28.9 ± 0.921	17.5 ± 0.927	18.6 ± 0.931
**145**	23.3 ± 0.304	21.7 ± 0.402	16.9 ± 0.501	19.2 ± 0.497
**146**	44.0 ± 0.057	45.5 ± 0.666	39.9 ± 0.618	47.6 ± 1.06
**147**	18.3 ± 0.373	17.4 ± 0.619	15.3 ± 0.469	19.5 ± 1.33
**148**	>70	>70	>70	>70
**149**	19.6 ± 1.60	22.0 ± 0.546	16.0 ± 0.368	17.0 ± 0.377
**150**	15.0 ± 0.689	15.0 ± 0.363	14.2 ± 0.670	13.7 ± 1.25
**151**	46.7 ± 1.90	46.2 ± 0.697	45.5 ± 1.04	46.9 ± 0.230
**152**	46.1 ± 0.653	45.2 ± 1.27	41.3 ± 0.346	44.2 ± 0.280
**153**	19.0 ± 1.13	22.5 ± 0.606	17.8 ± 0.193	16.6 ± 0.591
**154**	34.5 ± 0.187	39.5 ± 1.05	30.7 ± 0.480	27.3 ± 0.338
**155**	41.5 ± 1.83	43.2 ± 1.61	38.4 ± 1.15	38.5 ± 0.956

**Table 14:** Cytotoxicity (ED_50_ values in μM) of **156**–**166** in a panel of various cancer cell lines.

Compound	KB	KB-VIN	A549	1A9	HCT-8	ZR-751	PC-3	DU-145	LN-Cap
**GA**	>21	>21	NA	>21	19.5	NA	>21	>21	>21
**DZ**	NA	NA	>52	33.9	>52	>52	>52	>52	51
**156**	1.6	2.5	2	0.9	1.7	2.8	1.4	3.1	0.6
**157**	0.8	2.8	2.2	0.8	1.9	3	1.1	3.6	2.8
**158**	0.9	1.9	2.8	1.6	2	1.9	2.8	9.9	6.5
**159**	6.2	>15	15.5	5.9	2.6	>15	7.4	>15	1.9
**160**	1.8	1.7	1.7	1.1	2.7	5.2	3.3	5.8	1.1
**161**	2.9	13.2	3	1.8	4.9	8.8	3.5	>15	6.8
**162**	3	8.7	3.2	1.3	2.2	2.7	1.6	2.7	4.4
**163**	NA	NA	>14	>14	>14	NA	>14	>14	>14
**164**	9.9	NA	>14	13.3	>14	>14	14.1	>14	14.1
**165**	NA	NA	NA	>14	>14	NA	14.1	>14	14.1
**166**	>14	>14	NA	NA	>14	NA	>14	13	>14

**Table 15:** Cytotoxicity (IC_50_ values in μM) of **167**–**175** in a panel of various cancer cell lines.

Compound	518A2	8505C	A253	A549	DLD-1	Lipo	SW1736
**GA**	83.92	86.50	80.78	82.76	81.21	81.44	76.93
**167**	>30	>30	>30	>30	>30	>30	>30
**168**	>30	>30	>30	>30	>30	＞30	>30
**169**	>30	>30	>30	>30	>30	>30	>30
**170**	15.19	15.59	15.89	20.27	22.98	15.46	19.87
**171**	28.99	>30	>30	>30	>30	>30	28.64
**172**	21.00	8.82	10.97	4.28	23.09	11.47	1.88
**173**	14.91	11.61	13.57	19.16	14.88	12.77	16.36
**174**	15.33	15.59	15.89	20.27	22.98	15.46	19.87
**175**	>30	>30	>30	>30	>30	>30	>30

**Table 16:** Cytotoxicity (IC_50_ values in μM) of **176**–**199** in a panel of various cancer cell lines.

Compound	MCF-7	MDA-MB-231	hTERT-RPE1
**GA**	75.66 ± 1.52	84.70 ± 1.73	63.41 ± 1.07
**176**	13.64 ± 0.93	5.03 ± 0.82	17.32 ± 1.21
**177**	22.46 ± 1.26	8.14 ± 0.76	22.80 ± 0.97
**178**	20.29 ± 1.47	14.38 ± 0.52	29.63 ± 1.16
**179**	24.45 ± 1.36	14.46 ± 0.58	28.41 ± 0.87
**180**	8.54 ± 0.67	7.31 ± 0.16	18.59 ± 0.54
**181**	19.27 ± 1.01	9.41 ± 1.03	21.11 ± 0.73
**182**	14.90 ± 0.75	20.84 ± 1.20	24.09 ± 0.88
**183**	19.30 ± 0.98	23.15 ± 1.07	22.88 ± 0.68
**192**	6.00 ± 0.43	3.52 ± 0.61	10.36 ± 0.80
**193**	1.88 ± 0.20	1.37 ± 0.18	4.93 ± 0.36
**194**	8.62 ± 0.23	5.36 ± 0.44	16.28 ± 0.51
**195**	8.45 ± 0.32	3.49 ± 0.61	12.33 ± 0.46
**196**	7.24 ± 0.30	6.43 ± 0.84	8.48 ± 0.73
**197**	6.02 ± 0.35	6.27 ± 0.24	6.33 ± 0.19
**198**	2.65 ± 0.12	2.31 ± 0.65	5.65 ± 1.02
**199**	2.42 ± 0.23	1.86 ± 0.29	7.08 ± 0.73

**Table 17:** Cytotoxicity (IC_50_ values in μM) of **200**–**206**, **209** and **210** in a panel of various cancer cell lines.

Compound	HepG2	DU-145	MDA-MB-231
**GA**	74.35 ± 2.03	69.40 ± 2.37	72.65 ± 1.67
**200**	>100	21.59 ± 3.22	24.66 ± 2.71
**201**	>100	>100	89.40 ± 2.85
**202**	>100	>100	>100
**203**	10.01 ± 2.29	11.96 ± 1.42	17.80 ± 1.76
**204**	>100	>100	79.3 ± 2.34
**205**	36.37 ± 1.89	>100	40.65 ± 2.11
**206**	>100	>100	>100
**209**	>100	>100	>100
**210**	>100	>100	>100

## Abbreviations

DCC	Dicyclohexylcarbodiimide
DCM	Dichloromethane
DEAD	Diethyl azodicarboxylate
DMAP	4-Dimethylaminopyridine
DMF	*N*,*N*-Dimethylformamide
DMSO	Dimethylsulfoxide
EDCI	1-Ethyl-3-(3-dimethylaminopropyl)carbodiimide hydrochloride
HMPT	Hexamethylphosphoryl triamide
HOBt	1-Hydroxybenzotriazole
IBX	2-Iodoxybenzoic acid
*m*-CPBA	*m*-Chloroperbenzoic acid
NMP	*N*-Methylpyrrolidone
*p*-TSA	*p*-Toluenesulfonic acid
TFA	Trifluoroacetic acid
THF	Tetrahydrofuran
TMSOTf	Trimethylsilyltrifluoro methanesulfonate
